# Genome-Wide Identification of the WRKY Gene Family in Four Cotton Varieties and the Positive Role of *GhWRKY31* in Response to Salt and Drought Stress

**DOI:** 10.3390/plants13131814

**Published:** 2024-07-01

**Authors:** Tianyu Dong, Jiuchang Su, Haoyuan Li, Yajie Du, Ying Wang, Peilei Chen, Hongying Duan

**Affiliations:** 1 College of Life Sciences, Henan Normal University, Xinxiang 453007, China; dongty536@163.com (T.D.); 2021034@htu.edu.cn (J.S.); 2104283124@stu.htu.edu.cn (H.L.); gehtbbrt@163.com (Y.D.); wangy32021@163.com (Y.W.); chenpeilei@htu.edu.cn (P.C.); 2 Henan International Joint Laboratory of Aquatic Toxicology and Health Protection, College of Life Science, Henan Normal University, Xinxiang 453007, China

**Keywords:** WRKY, transgene, gene silencing, molecular docking, yeast one-hybrid

## Abstract

The WRKY gene family is ubiquitously distributed in plants, serving crucial functions in stress responses. Nevertheless, the structural organization and evolutionary dynamics of WRKY genes in cotton have not been fully elucidated. In this study, a total of 112, 119, 217, and 222 WRKY genes were identified in *Gossypium arboreum*, *Gossypium raimondii*, *Gossypium hirsutum*, and *Gossypium barbadense*, respectively. These 670 WRKY genes were categorized into seven distinct subgroups and unequally distributed across chromosomes. Examination of conserved motifs, domains, *cis*-acting elements, and gene architecture collectively highlighted the evolutionary conservation and divergence within the WRKY gene family in cotton. Analysis of synteny and collinearity further confirmed instances of expansion, duplication, and loss events among WRKY genes during cotton evolution. Furthermore, *GhWRKY31* transgenic *Arabidopsis* exhibited heightened germination rates and longer root lengths under drought and salt stress. Silencing *GhWRKY31* in cotton led to reduced levels of ABA, proline, POD, and SOD, along with downregulated expression of stress-responsive genes. Yeast one-hybrid and molecular docking assays confirmed the binding capacity of GhWRKY31 to the W box of *GhABF1*, *GhDREB2*, and *GhRD29*. The findings collectively offer a systematic and comprehensive insight into the evolutionary patterns of cotton *WRKYs*, proposing a suitable regulatory framework for developing cotton cultivars with enhanced resilience to drought and salinity stress.

## 1. Introduction

The increase in worldwide temperatures presents hurdles for the growth and maturation of higher plants, as they encounter diverse abiotic stresses such as extreme temperatures, drought, and high salinity. These stresses not only hinder plant growth but also contribute to a gradual decline in global crop production [1,2,3]. To address these challenges and acclimate to adverse growth conditions, plants have evolved a series of intricate regulatory mechanisms [4,5]. In this complex system, stress receptor genes, stress-related transcription factors (TFs), and downstream response genes collaborate to create a sophisticated interconnected network [6,7,8,9].

The transcription factors (TFs), which play vital roles in regulating gene expression by binding to *cis*-acting elements upstream of the transcription start site (TSS), are critical components in the molecular network of stress response [10,11,12,13]. WRKY TFs are one of the most extensive in plants, playing crucial roles in diverse responses to abiotic stressors [14,15]. The WRKY family members have a highly conserved WRKY domain, consisting of the WRKYGQK motif and a CX_4–5_CX_22–23_HXH zinc-finger motif [16]. The WRKYGQK motif binds to the W box (TTGACC/T) on the promoter of downstream genes and regulates their expression under various abiotic stresses [17,18]. WRKY TFs are typically classified into seven groups based on the number of WRKY domains (two domains in Group I proteins and one in the others) and the primary amino acid sequence (C_2_-H_2_ structure in Group IIa-e proteins and C_2_HC structure in Group III proteins) [16,18].

Throughout the growth and development of higher plants, encountering stress is unavoidable, necessitating a robust defense mechanism to mitigate its impact. WRKY TFs associated with plant responses to abiotic stress have been extensively documented. For instance, *AtWRKY46* displays rapid induction under water stress conditions and regulates genes involved in reactive oxygen species (ROS) scavenging and cellular osmoprotection. Overexpression of *AtWRKY46* leads to heightened sensitivity to osmotic stress in soil-grown *Arabidopsis* [19]. Conversely, silencing *AtWRKY63* suppresses the expression of stress-responsive genes *RD29A* and *COR47*, thereby compromising drought tolerance in *Arabidopsis* seedlings [20]. The double mutants of *AtWRKY25* and *AtWRKY33* in *Arabidopsis* exhibit heightened susceptibility to salt stress, whereas overexpression of either *AtWRKY25* or *AtWRKY33* enhances salt stress tolerance [21]. The heterologous expression of wheat *TaWRKY146* in *Arabidopsis* enhances drought resistance by facilitating stomatal closure, elevating proline levels, and reducing malondialdehyde (MDA) accumulation [22]. In *Camellia sinensis*, upregulation of *CsWRKY2* under exogenous abscisic acid (ABA) and drought stress enhances drought tolerance by modulating downstream genes in the ABA signaling pathway [23]. Moreover, overexpression of *DgWRKY2*/*3*/*4* significantly promotes germination rate and root length in soybean and *Arabidopsis* seedlings under high salinity conditions [24,25,26]. Collectively, these findings underscore the pivotal role of WRKY family members as key regulators in plants under drought and salt stress scenarios [27].

Cotton, the most vital oilseed and fiber crop, accounts for 35% of the total fiber used worldwide. Previous studies have suggested that all diploid and tetraploid cotton species have evolved from a common ancestor, which subsequently diversified to produce nine groups, including the A-G, K, and AD genomes [28,29]. Cultivated cotton mainly consists of four cotton subspecies, including *G. arboretum* (A2-genome species), *G. raimondii* (D5-genome species), *G. hirsutum* (AD1-genome species), and *G. barbadense* (AD2-genome species). Encouragingly, due to the release of high-quality whole-genome sequences of the four cultivated species [30,31,32,33], the genome-wide analysis of the WRKY gene family is feasible and will help to elucidate its regulatory functions in stimuli responses such as osmotic, drought, and salt. Nevertheless, the precise and systematic investigation of WRKY genes in cultivated cotton is largely understudied, and the comprehensive functional validation of *WRKYs* in response to osmotic, drought, and salt stress remains incomplete [34].

In this work, the phylogenetic tree, chromosomal distribution, cis-acting elements, conserved motifs and domains, and collinearity relationship of *WRKYs* were analyzed in cotton. We next confirmed that *GhWRKY31* was up-regulated under salt and drought stress. Functional assays involving heterologous expression and VIGS revealed that *GhWRKY31* contributed to salt and drought tolerance in both *Arabidopsis* and cotton. The drought- and salt-induced expression of genes, such as *GhABF1*, *GhABF2*, *GhDREB2*, *GhRD29*, *GhNAC4*, *GhP5CS*, and *GhSOS1*, was inhibited in *GhWRKY31*-silencing seedlings. Furthermore, the YIH assay confirmed the binding of GhWRKY31 to the W box of *GhABF1*, *GhDREB2*, and *GhRD29*. Our results not only present a comprehensive analysis of the cotton WRKY gene family but also provide new insights for breeding cotton against abiotic stresses.

## 2. Results

### 2.1. Identification and Phylogenetic Analysis of the WRKY Gene Family

**Figure 1:**
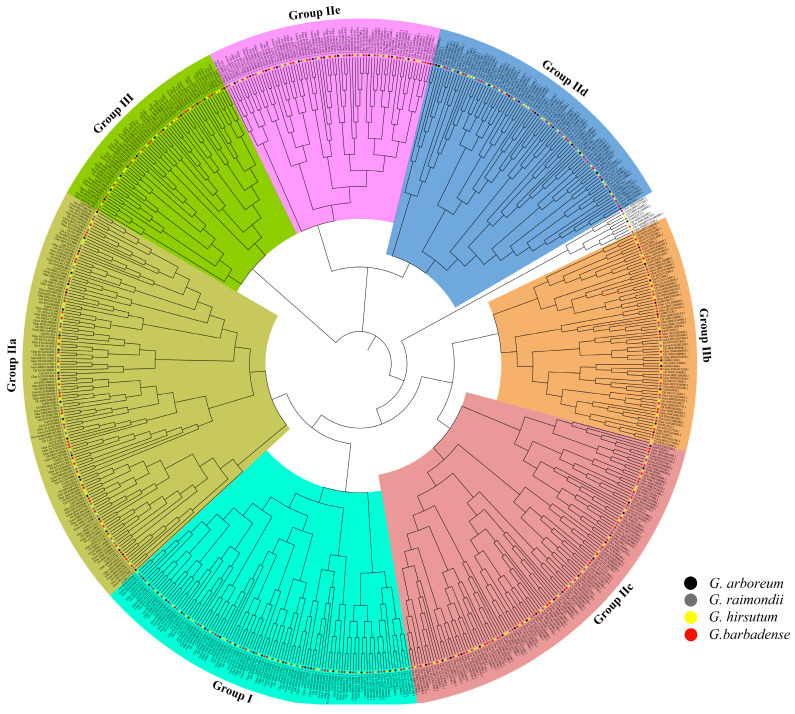
A maximum likelihood (1000 bootstraps) phylogenetic tree of WRKY proteins in *G. arboreum*, *G. raimondii*, *G. hirsutum*, and *G. barbadense*. The 7 color modules represent the 7 subfamilies of WRKY proteins, and no background module indicates unclassified WRKY proteins.

The shortest WRKY protein, Gbar_D06G009260, comprises 144 amino acids, whereas the longest proteins, Gbar_D12G019910 and Gorai.008G200800, consist of 1340 amino acids. The isoelectric point (pI) values vary from 4.72 (Gbar_D11G016820) to 9.98 (Gorai.004G219300 and Gh_D08G210300). The molecular weights (MW) range from 16,630.56 (Gorai.011G114200) to 151,574.34 (Gorai.008G200800). Subcellular localization analysis using the Plant-PLoc database indicated that WRKY proteins predominantly localize in the nucleus (Supplementary Table S1). A phylogenetic tree was constructed by employing the maximum likelihood method to elucidate the evolutionary relationships among 670 WRKY proteins in cotton (Figure 1). The WRKY proteins were categorized into seven clades, encompassing Group I (107), Group IIa-IIe (134, 74, 122, 86, and 75), and Group III (63), which were further distributed unevenly across seven subgroups.

### 2.2. Chromosome Location of WRKY Genes

**Figure 2:**
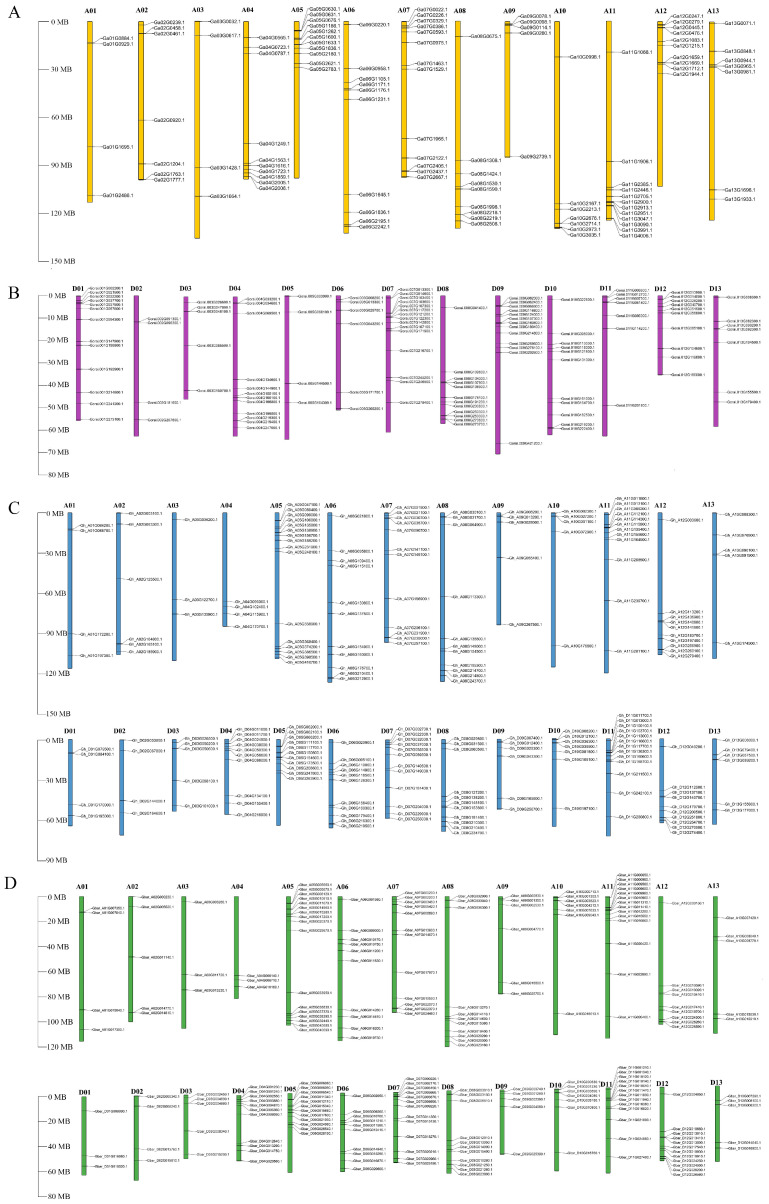
Chromosomal distribution of WRKY genes in (**A**) *G. arboretum*, (**B**) *G. raimondii*, (**C**) *G. hirsutum*, and (**D**) *G. barbadense*. The chromosome number is shown at the top of each chromosome, and the scale for the length of chromosomes is megabases (Mb).

To further analyze the distribution of WRKY genes, the chromosome location was mapped (Figure 2). In *Gossypium arboretum* and *Gossypium raimondii*, 109 and 119 WRKY genes were, respectively, localized in the At or Dt sub-genomes. Ga14G1656, Ga14G1714, and Ga14G1560 were identified within contigs (Figure 2A,B). The highest number of WRKY genes in *G. arboreum* was found on the A07 chromosome (13), whereas the lowest was on the A03 chromosome (4) (Figure 2A). For *G. raimondii*, the D01 and D09 chromosomes harbored the highest number of *GrWRKYs* (13), whereas the D02 and D05 chromosomes contained the fewest (4) (Figure 2B). In *Gossypium hirsutum*, chrA05 exhibited the highest WRKY gene count with 16 members, whereas chrA03 had the lowest with three *GhWRKYs*. Notably, Gh_Contig00579G000600, Gh_Contig00383G000300, and Gh_Contig01109G001300 were located in contigs without chromosomal assignments (Figure 2C). In *Gossypium barbadense*, WRKY genes were mapped across chrA01 to chrA13 (four to seventeen genes per chromosome) and chrD01 to chrD13 (three to thirteen genes per chromosome) (Figure 2D). Consequently, WRKY family members were distributed disparately among cotton chromosomes.

### 2.3. Conserved Motifs and Domains, Cis-Acting Elements, and Gene Structure of WRKYs

**Figure 3:**
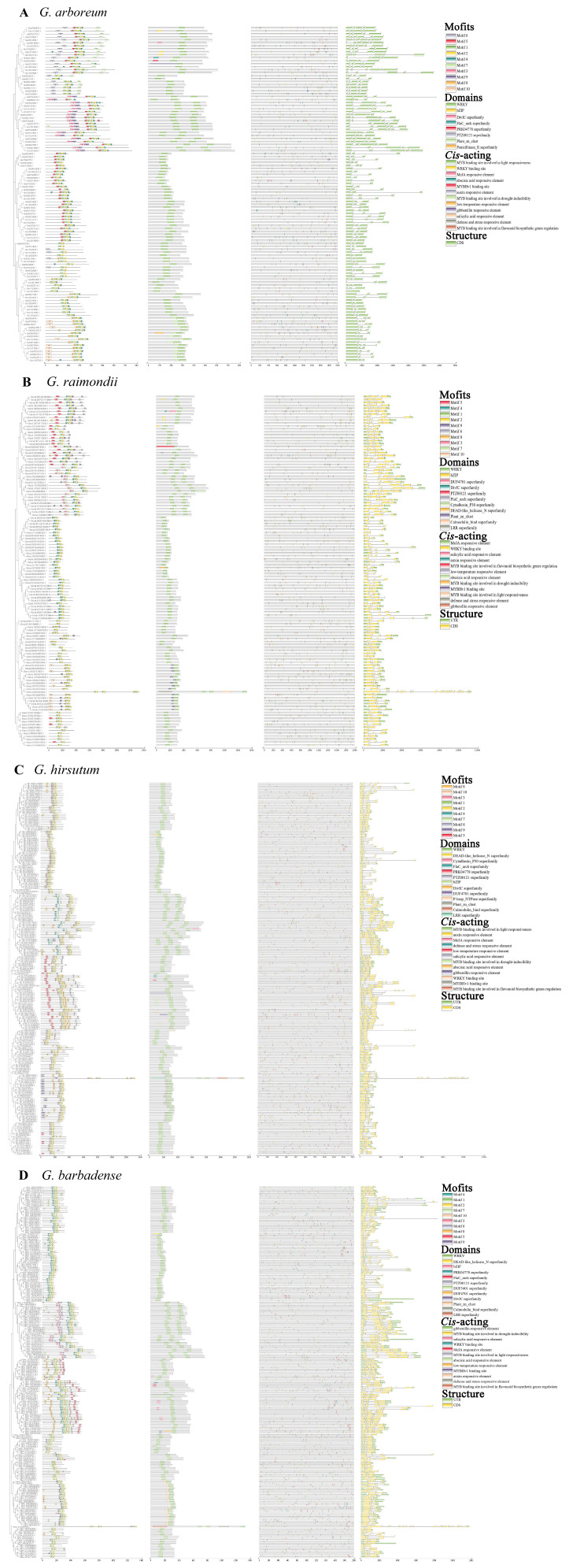
Analysis of conserved motifs and domains, *cis*-acting elements, and structures of WRKY members in (**A**) *G. arboreum*, (**B**) *G. raimondii*, (**C**) *G. hirsutum*, and (**D**) *G. barbadense*. The identification elements are represented by distinct colored boxes. The black lines of the gene structure indicate non-conserved regions.

To elucidate the detailed characteristics of *WRKYs*, an analysis encompassing gene structure, conserved motifs, domains, and *cis*-acting elements was conducted. Ten conserved motifs were identified in WRKY proteins across four cotton strains. Notably, the majority of WRKY proteins exhibited more than two motifs, with exceptions noted in five GaWRKY, 15 GrWRKY, and 30 GbWRKY proteins. Consistently, motifs 1 and 2 were universally present in all WRKY members (Supplementary Table S2). Furthermore, the identification of at least one WRKYGQK domain in WRKY proteins was observed, with 107 Group I WRKY proteins containing two WRKYGQK domains (Figure 3). Additionally, the presence of basic region-leucine zipper (bZIP) domains (PF00170) was detected in 20 WRKY proteins, and plant_zn_clust (PF10533) structures were predominantly situated at the N-terminus of 80 WRKY members (Figure 3). These findings underscore the evolutionary stability and diversity of WRKY proteins within the cotton genome. 

To further investigate the biological function of *WRKYs*, we identified *cis*-acting elements in the 5′-upstream regions of 2000 bp (Supplementary Table S3). A total of 12 different functions of *cis*-elements were identified, and these *cis*-acting elements related to stress responses were found abundantly in the promoter of *WRKYs* (Figure 3). The *cis*-acting elements can be divided into three categories: hormone-responsive sites (auxin-responsive element, gibberellin-responsive element, and MeJA-responsive element), transcription factor binding sites (MYB binding site, MYBHv1 binding site, and WRKY binding site), and growth and development sites (MYB binding site involved in drought inducibility, light responsiveness, flavonoid biosynthesis, low-temperature-induced responses, and defense- and stress-induced responses) (Figure 3).

### 2.4. Duplication and Collinearity of GaWRKYs, GrWRKYs, GhWRKYs, and GbWRKYs

**Figure 4:**
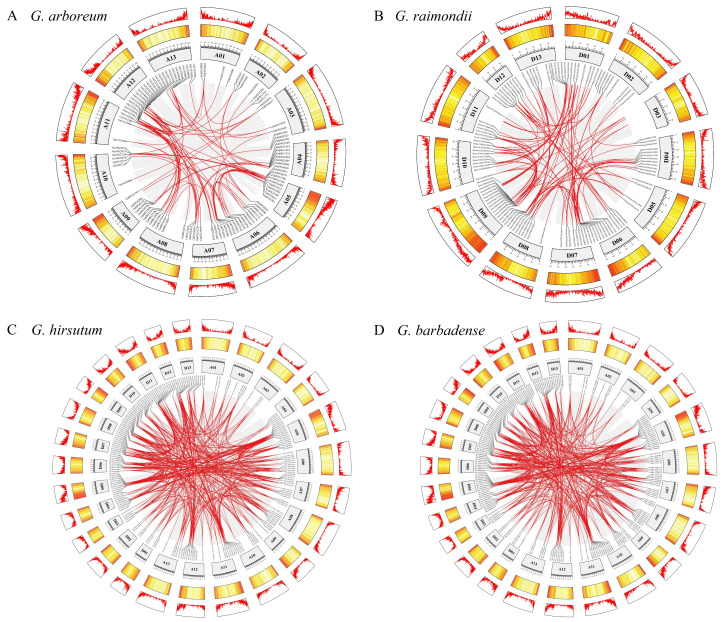
Duplicated WRKY genes based on the collinearity of all chromosomes in (**A**) *G. arboreum*, (**B**) *G. raimondii*, (**C**) *G. hirsutum*, and (**D**) *G. barbadense*. The number of genes is presented by a heatmap and a linear map, of which the red presents regions of high gene density, and yellow indicates a low-density region. The WRKY gene pairs with a syntenic relationship are linked by red lines, and the scale on the boxes above is in megabases (Mb).

The expansion pattern of *WRKYs* was elucidated through the construction of a duplication circos plot. In the diploid genomes of Gossypium raimondii and *Gossypium arboretum*, 88 and 102 *WRKYs*, respectively, were identified as originating from whole-genome duplication (WGD) or segmental duplication events (Figure 4A,B). Ga05G0631, Ga08G2219, Gorai.001G037800, Gorai.004G219400, and Gorai.009G062400 were tandem duplications and were distributed on chromosomes A05, A08, D01, D04, and D09, respectively (Figure 4A,B). Moreover, 17 and 14 WRKY genes were dispersed within the At or Dt sub-genomes (Supplementary Table S4). In *Gossypium hirsutum*, a substantial proportion (97.66%) of *WRKYs* were attributed to WGD or segmental duplication events. Among these, the WRKY genes Gh_A08G214800, Gh_D05G062100, and Gh_D08G210400 underwent tandem duplication, while Gh_D04G011700 and Gh_D07G055500 were dispersed, with respective locations on chromosomes A08, D04, D05, D07, and D08 (Figure 4C) (Supplementary Table S4). A total of 213 *WRKYs* had undergone whole-genome duplication (WGD) or segmental duplication events in *G. barbadense*. Gbar_A08G020300 (ChrA08), Gbar_D08G021260 (ChrD08), Gbar_A03G013230 (ChrA03), and Gbar_A11G020420 (ChrA11) appeared as tandem duplications or dispersion (Figure 4D) (Supplementary Table S4).

**Figure 5:**
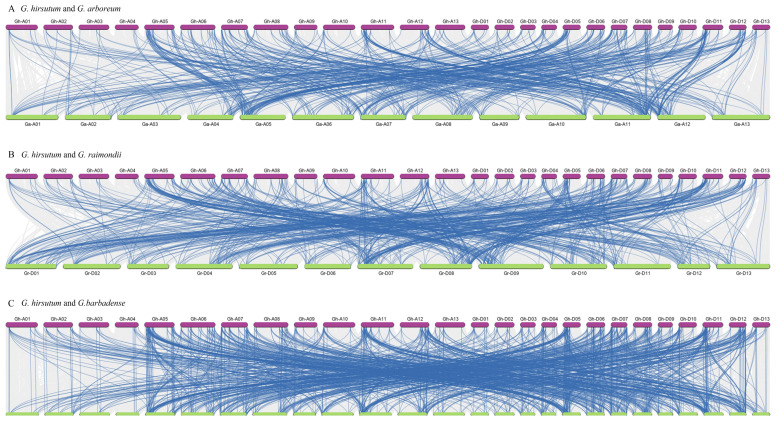
Synteny analysis of WRKY genes. Orthologous relationships between (**A**) *G. hirsutum* and *G. arboretum*, (**B**) *G. hirsutum* and *G. raimondii*, and (**C**) *G. hirsutum* and *G. barbadense* were investigated. Blue lines highlight duplicated WRKY gene pairs, while the gray lines in the background indicate all collinear relationships.

The hybridization event leading to the evolution of *Gossypium hirsutum* and *Gossypium barbadense* involved *Gossypium arboreum* (an A-genome species) and *Gossypium raimondii* (a D-genome species). A syntenic map was generated to investigate the evolutionary connections of WRKY genes between *G. hirsutum* and three other species (Supplementary Table S5). Through MCScan analysis, 571, 621, and 1044 duplicated gene pairs were identified between *G. hirsutum* and *G. arboreum*, *G. hirsutum* and *G. raimondii*, and *G. hirsutum* and *G. barbadense*, respectively (Figure 5). Notably, in *G. arboreum* and *G. raimondii*, the highest number of collinear relationships occurred on ChrA11 (85) and ChrD07 (98), with ChrA09 and ChrD05 exhibiting the fewest collinear relationships at 17 and 21, respectively (Figure 5A,B). Additionally, *G. barbadense* displayed 87, 75, 73, 65, and 78 collinear relationships on chromosomes A05, A11, D05, D07, and D11 among the 1044 gene pairs (Figure 5C). In short, the aforementioned results indicate an uneven distribution of collinear relationships across chromosomes, suggesting occurrences of deletion and duplication events within the WRKY gene family.

### 2.5. Expression Profiling and qRT-PCR Verification of GhWRKY Responses to Salt and Drought Stress

**Figure 6:**
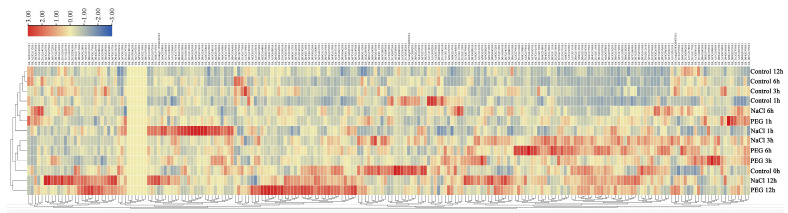
A cluster heatmap of expression patterns of *GhWRKYs* in response to NaCl and PEG treatment. Each line represents the expression of WRKY genes in different treatments, and the expression values in the row scale were normalized. The color scale varies from red to blue, indicating the high or low expression of each WRKY gene.

Transcriptome analysis of *Gossypium hirsutum* revealed distinct expression patterns of *GhWRKYs* under salt and drought stress conditions. Notably, following 3 h of salt treatment and 3 to 6 h of PEG treatment, a cluster of WRKY genes, such as Gh_A05G156700.1, Gh_D03G026500.1, Gh_A05G368400.1, and Gh_A06G109400.1, displayed elevated expression levels. Moreover, Gh_A05G156700.1, Gh_D02G067800.1, Gh_A08G149000.1, Gh_D08G210300.1, and Gh_D08G191400.1 exhibited peak expression levels after 1 and 6 h of salt treatment or 1 h of PEG treatment. Additionally, Gh_A08G031700.1, Gh_A09G013200.1, and Gh_D03G050200.1 reached their highest expression levels after 12 h of salt treatment or 12 h of PEG treatment. These findings indicate that a subset of *GhWRKYs* are responsive to salt and drought stress in *G. hirsutum* (Figure 6).

**Figure 7:**
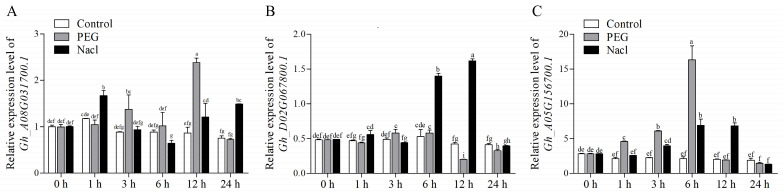
The expression levels of 3 WRKY genes in the leaves of *G. hirsutum* seedlings under PEG and NaCl stress. Gh_A08G037100.1 expression in the control group was set to 100% at 0 h. (**A**–**C**) represent the relative expression level of Gh_A08G031700.1, Gh_D02G067800.1, and Gh_A05G156700.1, respectively. Data represent the means ± SE from three independent experiments. The error bar represents the standard error of the mean, and the lowercase letter above the bar indicates a significant difference (*p* < 0.05).

Next, qRT-PCR was employed to assess the expression dynamics of selected *GhWRKYs* in response to PEG and NaCl treatments. As expected, Gh_A08G031700.1, Gh_D02G067800.1, and Gh_A05G156700.1 exhibited distinct expression patterns in the presence of PEG and NaCl solutions (Figure 7). Specifically, Gh_A08G031700.1 demonstrated sensitivity to both PEG and NaCl treatments, with upregulation observed at 3 and 12 h under PEG treatment, and at 1, 12, and 24 h under NaCl treatment (*p* < 0.05) (Figure 7A). Gh_D02G067800.1 responded primarily to salt stress, showing a significant increase in expression at 6 and 12 h under NaCl treatment (*p* < 0.05) (Figure 7B). Moreover, Gh_A05G156700.1 displayed enhanced expression levels at 1, 3, and 6 h following PEG treatment (*p* < 0.05), and similarly exhibited upregulation after NaCl treatment at 3, 6, and 12 h (Figure 7C). Thus, Gh_A05G156700.1 was chosen for subsequent functional validation studies under salt and drought stress conditions.

### 2.6. GhWRKY31 Improved the Tolerance of Transgenic Arabidopsis to Drought and Salt Stress

**Figure 8:**
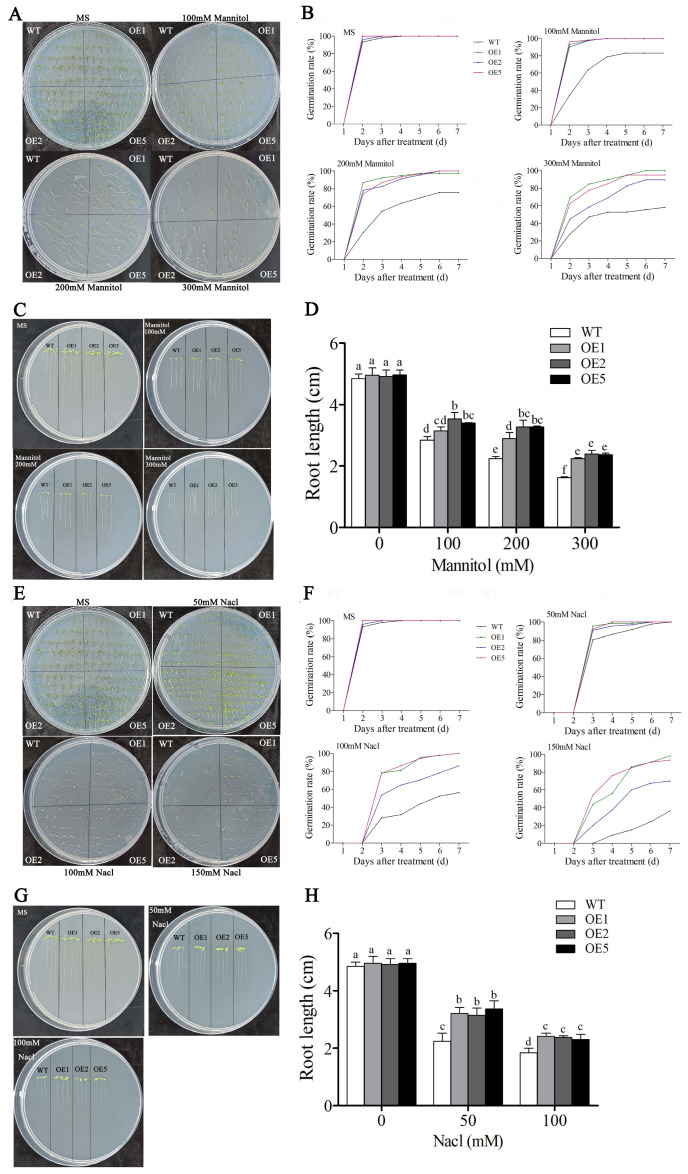
The germination rates and root length of *GhWRKY31* OE lines and WT under mannitol and salt conditions. (**A**,**E**) Phenotypic comparison of seedlings grown on 1/2 MS with 0 mM, 100 mM, 200 mM, and 300 mM mannitol or 0 mM, 50 mM, 100 mM, and 150 mM NaCl after 7 days. (**B**,**F**) Germination rates of seedlings grown under the conditions described in (**A**,**E**). (**C**,**D**,**G**,**H**) Phenotypic comparison and root length of seedlings grown on 1/2 MS with 0 mM, 100 mM, 200 mM, and 300 mM mannitol or 0 mM, 50 mM, and 100 mM NaCl after 7 days. Data represent the means ± SE from three independent experiments. The lowercase letters above the bar indicate the significant difference (*p* < 0.05).

qRT-PCR analysis confirmed the upregulation of *GhWRKY31* (Gh_A05G156700.1) in response to both drought and salt stress. Subsequently, a study was conducted to explore the role of *GhWRKY31* by assessing the drought and salt stress tolerance of transgenic *Arabidopsis* plants overexpressing *GhWRKY31* following homozygous molecular characterization (Supplementary Figure S1). Both seed germination and root length in WT *Arabidopsis* were significantly suppressed by mannitol and NaCl treatments. The germination rates of WT plants were notably reduced to 83% under 100 mM, 76% under 200 mM, and 58% under 300 mM mannitol treatment (Figure 8A,B), and were suppressed to 56% and 36% under 100 mM and 150 mM NaCl treatment, respectively (Figure 8E,F). Meanwhile, the root length of WT was also inhibited under 100 mM (2.84 cm), 200 mM (2.23 cm), and 300 mM (1.62 cm) mannitol (Figure 8C,D), and was suppressed to 2.24 and 1.84 cm under 50 mM and 100 mM salt conditions, respectively (Figure 8G,H). On the contrary, the germination rates and root length of *GhWRKY31* OE lines were significantly higher than those of WT. The germination rates were nearly 100%, 100%, and 90% under 100 mM, 200 mM, and 300 mM mannitol. Under 50 mM, 100 mM, and 150 mM NaCl solution, the germination rates of *GhWRKY31* OE lines were almost up to 100% (Figure 8B,F). In addition, the root length of OE lines was 3.36 cm, 3.14 cm, 2.33 cm, 3.24 cm, and 2.37 cm under 100 mM, 200 mM, 300 mM mannitol, 50 mM, and 100 mM NaCl treatments, respectively. These measurements were significantly longer than those of WT (*p* < 0.05) (Figure 8D,H). Hence, the heterologous expression of *GhWRKY31* in *Arabidopsis* significantly improved drought and salt tolerance.

### 2.7. VIGS of GhWRKY31 Reduced Drought and Salt Tolerance in G. hirsutum

**Figure 9:**
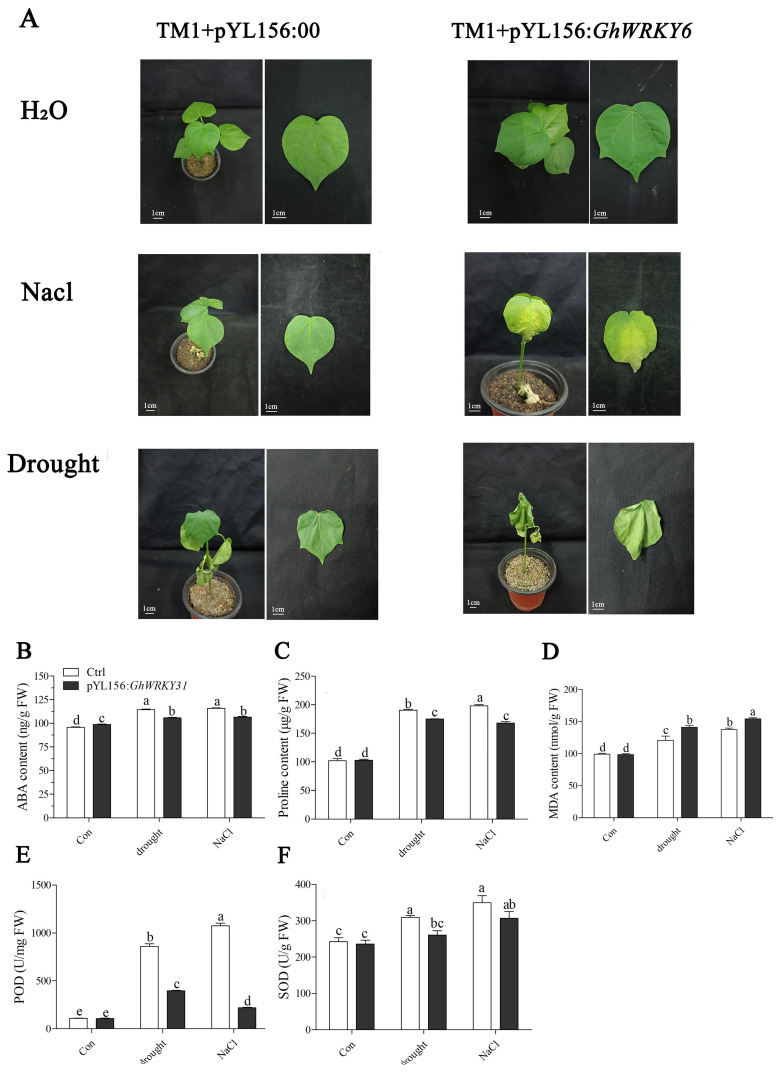
*GhWRKY31*-VIGS cotton seedlings exhibit increased sensitivity to drought and salt stress. (**A**) Leaf phenotypes showed shrinkage, yellowing, wilting, and death under water deficit conditions and 200 mM NaCl treatment. (**B**) ABA, (**C**) proline, (**D**) MDA content, (**E**) POD, and (**F**) SOD activity under water deficit conditions and 200 mM NaCl treatment. Data represent the means ± SE from three independent experiments. The lowercase letter above the bar indicates the significant difference (*p* < 0.05).

To further elucidate the function of *GhWRKY31* in *G. hirsutum*, VIGS was employed to decrease the transcription level of *GhWRKY31*. qRT-PCR was used to evaluate the silencing efficiency of *GhWRKY31*. The expression level of *GhWRKY31* was reduced by approximately 75% in pYL156: GhWRKY31 plants (Supplementary Figure S2). As expected, no stress-related phenotype was observed in the seedlings of ‘TM1+pYL156: 00’ and ‘TM1+pYL156: *GhWRKY31*’ under water conditions. Nevertheless, the leaves of ‘TM1+pYL156: *GhWRKY31*’ seedlings exhibited shrinkage and yellowing characteristics compared with ‘TM1+pYL156: 00’ (empty vector seedlings) under 200 mM NaCl treatment (Figure 9A). Meanwhile, after a 14-day water-deficit treatment, the leaves of ‘TM1+pYL156: 00’ showed a healthier phenotype compared to ‘TM1+pYL156: *GhWRKY31*’ seedlings. The latter exhibited symptoms such as shrinkage, rolling, wilting, and death (Figure 9A). Additionally, the ABA and proline contents accumulated less in ‘TM1+pYL156: *GhWRKY31*’ seedlings than in the control group seedlings under drought and salt stress. Meanwhile, MDA accumulation was higher in ‘TM1+pYL156: *GhWRKY31*’ seedlings compared to the seedlings in the control group. Moreover, the activities of peroxidase (POD) and superoxide dismutase (SOD) were higher in plants in the control group than in ‘TM1+pYL156: *GhWRKY31*’ plants under drought and salt stress (Figure 9B–F).

### 2.8. GhWRKY31 Regulates the Expression of Salt- and Drought-Induced Genes

**Figure 10:**
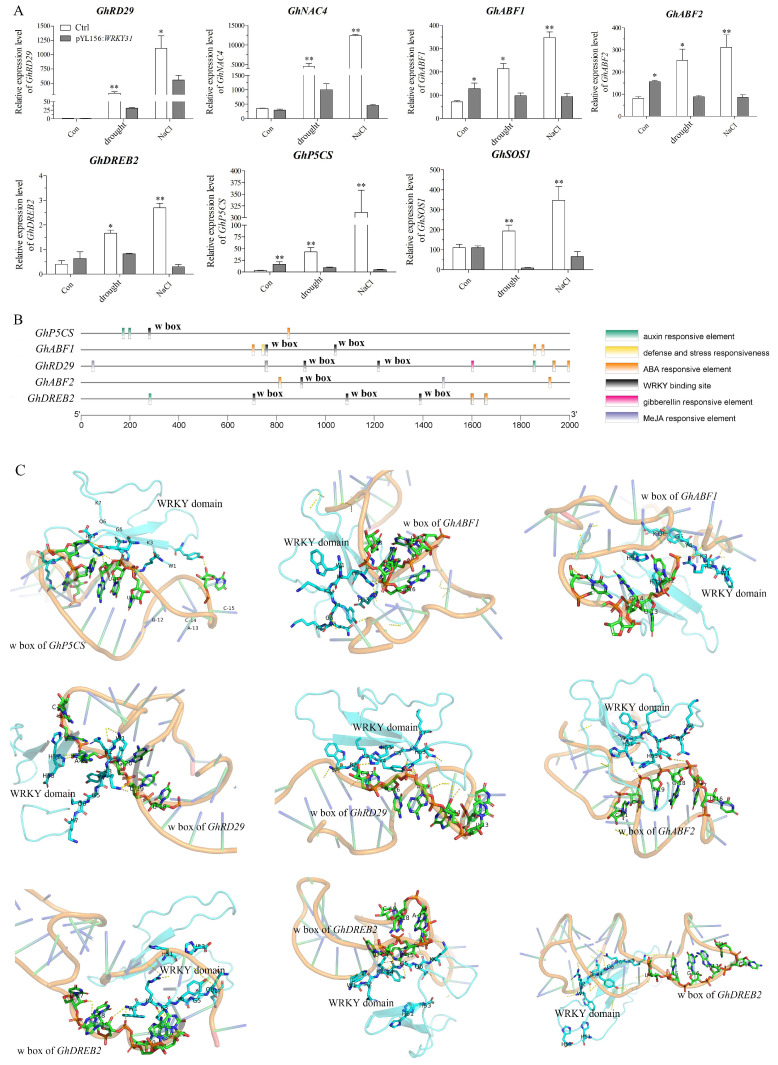
The gene expression levels induced by salt and drought were regulated by *GhWRKY31* in *G. hirsutum* leaves. (**A**) Silencing of *GhWRKY31* inhibits salt- and drought-induced gene expression. The data are shown as the mean ± SD from three independent biological replicates. (**, *p* < 0.01; *, *p* < 0.05; Student’s *t*-test). (**B**) The *cis*-acting elements are located 2000bp upstream of the *GhP5CS*, *GhABF1*, *GhRD29*, *GhABF2*, and *GhDREB2* promoters. (**C**) The 3D structure of molecular docking for the binding of the GhWRKY31 protein and the W boxes of *GhP5CS*, *GhABF1*, *GhRD29*, *GhABF2*, and *GhDREB2*. The yellow dashed line represents hydrogen bonding interactions.

The *GhWRKY31*-silenced cotton seedlings exhibited heightened sensitivity to drought and salt stress. To elucidate the target genes of *GhWRKY31* in response to drought and salt in cotton, we conducted qRT-PCR analysis to determine whether *GhWRKY31* is essential for the expression of ABA-, drought-, and salt-induced genes. The expression levels of *GhRD29*, *GhNAC4*, *GhABF1*, *GhABF2*, *GhDREB2*, *GhP5CS*, and *GhSOS1* were induced in the control group under drought and NaCl stress. However, silencing *GhWRKY31* resulted in a decrease in the induction of the seven genes under drought and salt stress. Specifically, the expression levels of *GhABF1*, *GhABF2*, *GhP5CS*, and *GhSOS1* were suppressed to levels lower than those observed in the control group (Figure 10A).

Further analysis focused on the identification of the W box (TTGACC/T) motif, which is crucial for the specific DNA binding of WRKY family members in the promoter regions of the aforementioned seven genes. We found that one, two, two, one, and three W boxes (TTGACC) were located in the promoter regions of *GhP5CS*, *GhABF1*, *GhRD29*, *GhABF2*, and *GhDREB2*, respectively (Figure 10B). Subsequently, molecular docking studies were performed using HDOCK v1.1 and PyMOL 2.5.0 software to explore potential interaction sites between the GhWRKY31 protein and the W box motifs of these five genes. The confidence scores for the interactions of GhWRKY31 with *GhP5CS*, *GhABF1*, *GhRD29*, *GhABF2*, and *GhDREB2* were determined as 0.8611, 0.9525/0.9050, 0.7619/0.8815, 0.8930, and 0.8576/0.8654/0.9492, respectively. The results indicated the formation of stable complexes between the WRKY domain of GhWRKY31 and the adjacent W box sequences, sustained by robust hydrogen bond interactions (Figure 10C).

### 2.9. GhWRKY31 Binds to the Promoter Regions of GhABF1, GhDREB2, and GhRD29

**Figure 11:**
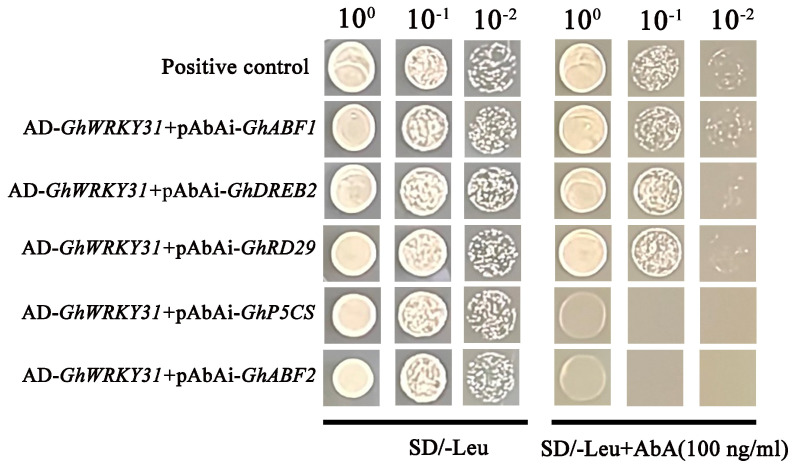
Y1H assay of GhWRKY31 with *GhABF1*, *GhDREB2*, *GhRD29*, *GhP5CS*, and *GhABF2*. The promoters of *GhABF1*, *GhDREB2*, *GhRD29*, *GhP5CS*, and *GhABF2*, which contain the putative TTGACC/T transformation (W box), were constructed in the pAbAi vector. The ORF of *GhWRKY31* was constructed in the pGADT7 vector. Yeast cells were diluted with distilled water (10^0^ to 10^−2^) and cultured on SD/-Leu medium supplemented with 100 ng/mL of Aureobasidin A (AbA).

The Yeast one-hybrid (Y1H) assay was employed to further investigate the binding affinity of the GhWRKY31 protein to *GhP5CS*, *GhABF1*, *GhABF2*, *GhDREB2*, and *GhRD29*. Firstly, we confirmed that 100 ng/mL of AbA could inhibit the self-activation of pAbAi-bait. The results showed that the transformation yeast containing the combination of GhWRKY31 with the W box (TTGACC/T) of *GhABF1*, *GhDREB2*, *GhRD29*, *GhP5CS*, and *GhABF2* grew on SD/-Leu medium. The GhWRKY31 protein specifically bound to the fragment that contained the core TTGACC/T motif of *GhABF1*, *GhDREB2*, and *GhRD29* in the SD/-Leu+AbA (100 ng/mL) medium. These findings support the conclusion that GhWRKY31 directly binds to the promoter regions of *GhABF1*, *GhDREB2*, and *GhRD29* (Figure 11).

## 3. Discussion

WRKY TFs are ubiquitously present throughout the plant kingdom, representing a remarkably conserved protein family. Currently, genome-wide studies of the WRKY gene family have been extensively conducted [14,15], revealing their crucial involvement in responding to various abiotic stressors [8,35,36]. Cotton, one of the most important economic crops, has remained relatively scarcely researched with regard to its WRKY gene family. Hence, the study investigates the evolution and function of WRKY genes in cotton based on analysis of genome-wide duplication, heterogenous expression in *Arabidopsis*, VIGS in *G. hirsutum* ‘TM1’, molecular docking, and Y1H.

In this study, 112 *GaWRKYs*, 119 *GrWRKYs*, 217 *GhWRKYs*, and 222 *GbWRKYs* were identified. Since cotton underwent hybridization and polyploidization 1.5 Mya, the number of WRKY genes in tetraploid cotton has increased to be ~2-fold greater than that of diploid cotton [37]. Next, the 670 *WRKYs* were divided into seven subgroups (Figure 1) based purely on phylogenetic data [38] and were unevenly distributed among different subfamilies. The analysis of chromosomal positioning showed the absence of WRKY genes on chrD01, chrD09, and chrD10, and the acquisition of WRKY genes on chrD05, chrD06, and chrD11 during the formation of tetraploid cotton. These findings provide valuable insights into the evolutionary dynamics of the WRKY gene family in cotton.

To further elucidate the evolutionary relationships among WRKY TFs in cotton, an analysis of the conserved motifs and domains of WRKY genes was conducted. Each WRKY gene typically exhibited one or two conserved WRKYGQK domains and a distinctive zinc-finger structure at the C-terminus, comprising consecutive conserved motifs (Figure 3). The WRKY domain was predominantly situated at the central region of the protein sequences, with consistent motifs and domains observed within the same subgroup across the four cotton species analyzed. These findings suggest a high degree of conservation among WRKY genes throughout cotton evolution. Nonetheless, sequence similarity in regions outside the motifs and domains of WRKY genes was comparatively lower, indicating complexity and diversity in the evolutionary trajectory of cotton WRKY genes (Figure 3). *Cis*-acting elements located in the promoter region are recognized for their crucial role in gene expression regulation, offering insights into gene functionality. The *cis*-acting elements of *WRKYs* encompass phytohormone response elements, development and stress-related elements, as well as transcription factor binding sites (Figure 3). These elements are likely to influence hormonal responses, abiotic stress responses, and interactions with transcription factors. Notably, similar *cis*-acting elements have been documented in other plant species such as *Vitis vinifera* [39], *Calohypnum plumiforme* [40], and *Chrysanthemum lavandulifolium* [41]. Furthermore, the structural analysis revealed significant variations in the proportions of UTRs and CDSs among the different cotton species, potentially attributed to homologous recombination events resulting from the artificial domestication of cotton [30,42].

In general, WGD, segmental duplication, tandem duplication, and transposon-induced duplication represent primary mechanisms capable of modifying the function, evolution, and configuration of TFs, leading to the emergence of novel subfamilies [43,44,45]. Our study revealed that the frequencies of WGD or segmental duplication events exceeded those of tandem duplication, underscoring the fact that the amplification and evolution of WRKY genes are predominantly driven by WGD or segmental duplication, with tandem duplication playing a secondary role (Figure 4). Likewise, these evolutionary events were also found in mung bean [10], wheat [15], and cherry [46], with WGD and segmental duplication events exerting a primary effect. In subsequent investigations into the evolutionary mechanisms of WRKY genes, synteny analysis revealed numerous collinear WRKY gene pairs between *G. hirsutum* and three other species (Figure 5). This conservation may be attributed to the stability in gene number and arrangement during the 1.5 million years of hybridization, polyploidy, and evolutionary processes in cotton. Nonetheless, partial WRKY genes were lost during evolution, potentially as a result of the artificial domestication process spanning 8000 years. The retention of these preserved WRKY genes is believed to significantly enhance cotton’s survival and adaptability, as well as the quality and length of its fibers [30,33].

Currently, WRKY TFs have been identified to play a crucial role in the regulation of plant responses to drought and salt stress [20,23,24,25]. Our investigations indicated that a cluster of WRKY genes exhibited differential expression in response to PEG or NaCl treatment (Figure 6), and the *GhWRKY31* emerged as a potential candidate gene associated with salt and drought stress response in *Gossypium hirsutum* (Figure 7). Subsequently, to delve deeper into the function of *GhWRKY31*, the Super1300: *WRKY31* vector was engineered, leading to the generation of homozygous *GhWRKY31* transgenic *Arabidopsis* lines. Assessment of phenotypic traits indicated that under stress conditions, the germination rate and root length of the WT were notably inferior to those of the *GhWRKY31* OE lines, suggesting that *GhWRKY31* was found to confer dual resistance to salt and drought stress in *Arabidopsis* (Figure 8). Similarly, the overexpression of *GhWRKY39-1* in *Nicotiana benthamiana* not only heightened salt stress tolerance but also conferred enhanced resistance to bacterial pathogen infection [47]. Transgenic tobacco overexpressing *GhWRKY25* exhibited improved seedling tolerance to salt stress while displaying decreased resistance to mannitol-induced osmotic and drought stress [48]. Furthermore, numerous WRKY genes have been documented to actively respond to osmotic, drought, and salt stress in various plant species. For example, the overexpression of *MbWRKY5* [49], *MfWRKY40* [50], *CmWRKY10* [51], and *TaWRKY93* [52] in *Arabidopsis* or tobacco led to heightened resistance to osmotic and high-salinity stress compared to the WT, whereas overexpression of *ZmWRKY17* [53], *CdWRKY50* [54], and *VvWRKY50* [55] resulted in susceptibility under PEG, mannitol, or NaCl treatment. These outcomes underscore the pivotal role of heterologous WRKY gene expression in diverse abiotic stress responses in *Arabidopsis* and tobacco.

**Figure 12:**
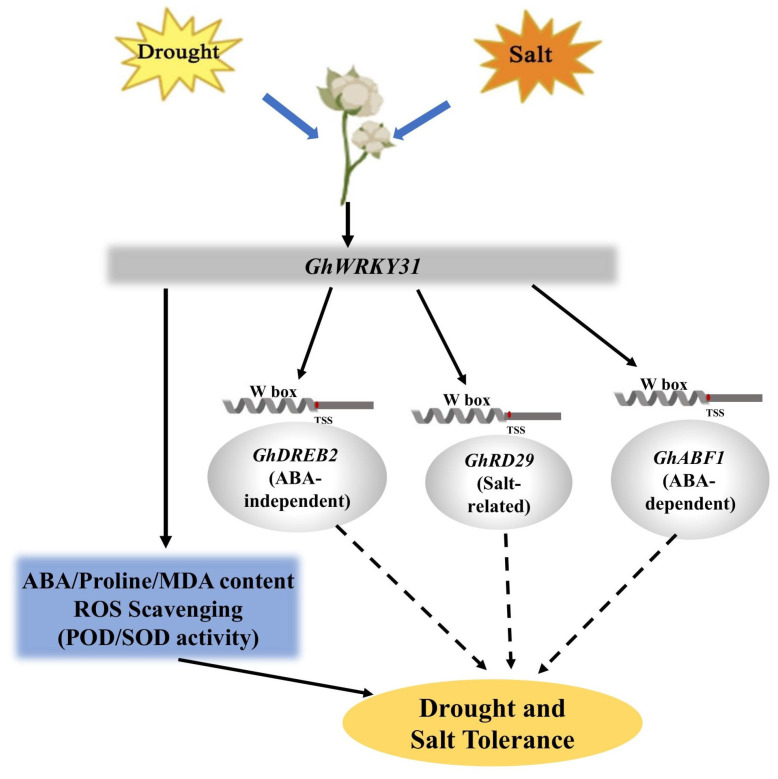
A working model of the role of the *GhWRKY31* module in drought and salt stress responses in cotton.

To enhance the understanding of *GhWRKY31*’s function, drought and salt tolerance assessments were conducted in *G. hirsutum* utilizing VIGS technology. The leaves of *GhWRKY31*-silenced cotton seedlings exhibited heightened sensitivity to water-deficit and NaCl conditions. Reduced levels of ABA and proline content, coupled with elevated MDA accumulation, indicated decreased resistance to drought and salt stress in VIGS cotton plants. Analysis of POD and SOD activities confirmed that the WT plants possessed superior ROS-scavenging capacity compared to *GhWRKY31*-silenced cotton seedlings (Figure 9). Notably, *ABF1*/*2*, *DREB2*, and *RD29* were identified as key players in ABA-dependent or ABA-independent responses to drought and salt stress, exerting a positive regulatory function under drought and NaCl conditions [56,57]. The induction of *P5CS*, a pivotal enzyme in proline biosynthesis, in response to drought and high salt levels was observed [58]. Our investigation revealed suppressed expression levels of *GhABF1*, *GhABF2*, *GhDREB2*, *GhRD29*, and *GhP5CS* in *GhWRKY31*-VIGS cotton leaves. Similarly, silencing of *GhWRKY46* [59] and *XsWRKY20* [60] via virus-induced gene silencing resulted in increased sensitivity to drought or salt stress, evidenced by weakened physiological phenotypes, heightened MDA content, diminished proline accumulation, and notable inhibition of stress-related gene expression levels, such as *ABI3*, *ABF2*, *DREB1*, *DREB2*, *RD22*, *LEA5*, and *P5CS*, in *WRKY*-silenced seedlings. Furthermore, molecular docking analysis illustrated the formation of stable complexes through multiple hydrogen bonds between the WRKYGQK domain of GhWRKY31 and the W boxes of *GhABF1*, *GhDREB2*, and *GhRD29* (Figure 10). Additional research has corroborated that the WRKYGQK domain of *SlWRKY3/4*, *CcWRKY1/51/70*, and *HvWRKY46* can establish hydrogen bonds with the W box of stress-related genes, exhibiting diverse bonding strengths among these members of the WRKY subfamily [61,62,63]. Additionally, Y1H analysis confirmed that *GhABF1*, *GhDREB2*, and *GhRD29* directly interact with the GhWRKY31 protein (Figure 11). Therefore, GhWRKY31, serving as a positive regulator in response to drought and salinity stress, has the potential to confer salt and drought resistance in *Arabidopsis* and cotton through the upregulation of *GhABF1*, *GhRD29*, and *GhDREB2* (Figure 12). Collectively, these findings not only support the advancement of research on WRKY genes implicated in stress resilience in cotton but also establish a theoretical framework for plant cultivation in arid and saline environments.

## 4. Materials and Methods

### 4.1. Identification of WRKY Family Members

Four cotton genome assembly files (FASTA format) and genome annotation files (GFF3 format), including *G. arboreum* (CRI version, strain SXY1) [30], *G. raimondii* (JGI version, strain Ulbr.) [31], *G. hirsutum* (CRI version, strain Tm-1) [32], and *G. barbadense* (HAU version, strain 3–79) [33], were downloaded from Cotton FGD [64]. The Hidden Markov Model (HMM) file (PF03106) was downloaded from the Pfam database (http://pfam.xfam.org/ (accessed on 22 April 2023)) [65]. HMMER 3.0 [66] was used to screen the potential WRKY proteins, and the key parameters were set as default (1 × 10^−5^). Next, WRKY proteins were manually screened using SMART (http://smart.emblheidelberg.de/ (accessed on 2 June 2023)) and NCBI CDD (https://www.ncbi.nlm.nih.gov/Structure/bwrpsb/bwrpsb.cgi/ (accessed on 3 June 2023)). Finally, non-WRKY domains and incorrect and repetitive family members were deleted.

### 4.2. Multiple Sequence Alignment and Phylogenetic Tree Construction

The full-length amino acid sequences of WRKY proteins were aligned using the ClustalW program. Based on the alignments provided, a maximum likelihood tree was constructed using the MEGA 7.0 program (http://www.megasoftware.net/ (accessed on 19 June 2023)) [67], and the bootstrap test was carried out with 1000 iterations. Finally, the phylogenetic tree was plotted using interactive tree of life v5.0 (iTOL) (https://itol.embl.de/ accessed on 25 June 2023)) [68].

### 4.3. Chromosomal Locations, Gene Structure, Conserved Motifs and Domains, and Cis-Acting Elements of WRKY Proteins

To map the chromosomal distribution of WRKY genes in 4 cotton species, the above reference genomes and annotation files, as well as WRKY protein IDs, were incorporated into the gene location visualization toolkit of TBtools [69]. For the analysis of WRKY gene structures, we extracted information on WRKY gene structures using reference genomes and annotation files. We then visualized the WRKY gene structures using the gene structure toolkit of TBtools. The conserved motifs of WRKY proteins were analyzed using the MEME database (http://meme-suite.org/ (accessed on 5 July 2023)), and the conserved domains were obtained through the NCBI CD-Search (https://www.ncbi.nlm.nih.gov/Structure/cdd/wrpsb.cgi (accessed on 8 July 2023)). The acquired data were then plotted using TBtools. To investigate the *cis*-acting elements of *WRKY* promoters, the 5′-upstream regions of 2000 bp were downloaded from Cotton FGD. Subsequently, the sequences were analyzed using the PlantCARE database (http://bioinformatics.psb.ugent.be/webtools/plantcare/html/ (accessed on 21 July 2023)) [70] and visualized with TBtools.

### 4.4. Duplication and Collinearity Analysis of WRKY Proteins

The MCScan program [71] was used to detect gene pairs with a BLASTp search (e-value < 10^−5^). Next, the chromosome length file, gene density file, and WRKY ID highlighting file were created from the reference genomes and annotation files, respectively. The prepared files were separately placed into the multiple collinearity scanning toolkit, dual synteny plotter toolkit, and advance circos toolkit of TBtools [72] for analysis of gene collinearity relationships and duplication events among the *WRKYs* in four cotton species.

### 4.5. Cotton Materials and Stress Treatments

Upland cotton *G. hirsutum* (Tm-1) was obtained from Cotton Research of the Chinese Academy of Agricultural Sciences (Anyang, Henan Province, China). The cotton seeds were sterilized with 3% H_2_O_2_ for 12 h and then washed with distilled water. Subsequently, the seedlings were grown in a greenhouse at 28 °C with a 16 h light/8 h dark photoperiod until the second true leaf expanded.

For the validation of expression levels for *GhWRKYs* under drought and salt stress treatments, cotton seedlings were irrigated with 20% (*w*/*v*) PEG6000 (drought-mimicking) and 200 mM NaCl solution, respectively. Seedlings with water were used as the control group. All leaves were collected at 0, 1, 3, 6, 12, and 24 h and stored at −80 °C for further experiments.

### 4.6. Transcriptome Analysis and qRT-PCR Verification of WRKY Genes

The RNA-seq raw data of PEG- and NaCl-treated *G. hirsutum* were downloaded from NCBI (https://www.ncbi.nlm.nih.gov/bioproject/?term=PRJNA490626 (accessed on 3 September 2023)) [73]. Firstly, we downloaded the raw sequencing data. Following the removal of adapters by Fastq and Trim Galore, the sequencing reads were aligned to the genome of *G. hirsutum* using STAR [74]. Next, RSEM [75] was employed to obtain the expression quantification (FPKM value) of *GhWRKYs*. The FPKM values of *WRKY* genes were log2-transformed and plotted using the TBtools heatmap.

The total RNA of *G. hirsutum* was extracted using a FastPure Universal Plant Total RNA Isolation Kit (Vazyme, Nanjing, China). The first-strand cDNA was synthesized using a TaKaRa kit (TaKaRa, Japan). RT-qPCR was performed using the Universal SYBR qPCR Master Mix kit (Vazyme, Nanjing, China), with 1 μL of cDNA template, 0.5 μL each of forward and reverse primers (at a working concentration of 10 μM), 5 μL of SYBR qPCR master mix, and 3 μL of nuclease-free water. The primers were designed using Primer Premier 5.0 (Supplementary Table S6). *GhActin7* was used as an internal control. A total volume of 10 μL was carried out in the Light Cycler^®^ 96 fluorescence quantitative PCR instrument (ABI7500; Applied Biosystems, America). The expression levels of WRKY genes were calculated by the 2^−∆∆Ct^ method [76].

### 4.7. Heterologous Overexpression and Stress Tolerance Assay in Arabidopsis

The wild-type (WT) *Arabidopsis* (Ecotype Col-0) was used as the receptor for *GhWRKY31* genetic transformation. The seeds of WT *Arabidopsis* were surface-sterilized with 5% sodium hypochlorite and washed with sterile water 5 times. These seeds were then stored at 4 °C for 24 h. Next, the seeds were evenly sown on 1/2 MS solid mediums and cultured in a greenhouse (16 h light/8 h dark cycle, 22 °C) for 7 days. Next, these seedlings were replanted in a 3:1 mixture of vermiculite and nutrient soil.

The *GhWRKY31* CDS was inserted into the *XbaI*/*KpnI* restriction enzyme sites of the Super1300 plasmid. The Super1300:*GhWRKY31* vector was transformed into *Agrobacterium tumefaciens* strain GV3101, and full-flowering *Arabidopsis* seedlings were used for genetic transformation by the floral dip method [77]. The *GhWRKY31* overexpression (OE) lines were selected using hygromycin. Here, the 4-week-old OE lines of *GhWRKY31* were identified by RT-qPCR. We finally obtained 5 independent OE lines of *GhWRKY31* and named them OE1, 2, 3, 4, and 5.

The seeds of WT and *GhWRKY31* OE lines of *Arabidopsis* were evenly planted on 1/2 MS solid media containing different concentrations of mannitol (0, 100, 200, and 300 mM) and NaCl (0, 50, 100, and 150 mM). The germination rate was recorded for 7 consecutive days using a magnifier. To measure root length, seedlings were initially grown upright on fresh 1/2 MS solid medium for 3 days, and then transferred to 1/2 MS solid medium supplemented with mannitol (0, 100, 200, and 300 mM) and NaCl (0, 50, and 100 mM) for a period of 5 to 7 days.

### 4.8. Virus-Induced Gene Silencing (VIGS), Stress Treatments, and Determination of Biochemical Indexes

As a previous study described [78], the CDS of *GhWRKY31* was amplified from *G. hirsutum* using RT-PCR. The CDS of *GhWRKY31* was inserted into the pYL156 vector to construct a pYL156: *GhWRKY31* fusion vector. Subsequently, the pYL156: 00 (empty vector) and pYL156: *GhWRKY31* vectors were severally transformed into *A. tumefaciens* strain GV3101. The bacterial fluid of recombinant GV3101 was used to infect the cotyledons of *G. hirsutum* seedlings through injection. The leaves were collected for RNA extraction and to detect interference efficiency using qRT-PCR.

For the drought and salt tolerance assays, seedlings of ‘TM1’+pYL156: 00 (empty vector injection) and ‘TM1’+pYL156: *GhWRKY31* (*GhWRKY31* injection) were treated for 14 days with water, water-deficit conditions, and 200 mM NaCl solution. Additionally, the cotton leaves were crushed and added to the cold alcohol extract. The ABA was dissolved from the plant cells into the extraction solution by stirring at a low temperature. A centrifuge was used to separate the plant residues and cell fragments suspended in the alcohol extract. The ABA concentrate was obtained by transferring the alcohol extract into a new centrifuge tube. Subsequently, ABA levels (ng/g. FW) were detected using an ABA Elisa kit (SIONBESTBIO, YX-E21782, Shanghai, China). Following the crushing of cotton leaves, proline and MDA extracts were added separately and the mixture was subjected to low-temperature stirring to ensure full contact. A centrifuge was employed to isolate plant residues and cell fragments that were suspended in the extract. Analysis of proline (μg/g. FW) (Solarbio, BC0290, Beijing, China) and MDA (nmol/g. FW) (Solarbio, BC0025, Beijing) contents was carried out using dedicated kits for each compound.

To determine SOD (U/g. FW) and POD (U/mg. FW) activity, cotton leaves were finely crushed and mixed with a test tube containing phosphoric acid buffer and polyethylene glycol /EDTA. The mixture was stirred under ice bath conditions to ensure complete contact between the sample and the extract, facilitating the release of the POD and SOD enzymes from the cells. The resulting extract was then centrifuged and transferred to a new centrifuge tube for activity determination using a POD kit (Solarbio, BC0095, Beijing, China) and a SOD kit (Solarbio, BC0175, Beijing, China).

### 4.9. Molecular Docking Simulation

The interaction between the GhWRKY31 protein and the W box of stress-related genes was investigated using HDOCK v1.1 software. The nucleotide sequence of stress-related genes and the amino acid sequence of GhWRKY31 were introduced into receptors and ligands modules in HDOCK (http://hdock.phys.hust.edu.cn/ (accessed on 6 January 2024)) [79]. The output interaction model files were imported into PyMOL 2.5.0. The center of the docking boxes, which were based on the position of the crystal ligand, were constructed minutely. Next, the atoms for polarity docking were selected, and the docking relationship was plotted. In addition, the confidence score (CS) indicates the likelihood of binding between two molecules (CS = 1.0/[1.0 + e^0.02×(docking score+150)^]). The two molecules would be very likely to bind if CS > 0.7.

### 4.10. Yeast One-Hybrid (Y1H) Assay

The Y1H assay was performed following the same methodology as a previous study [80]. The full-length sequence of *GhWRKY31* was cloned into the pGADT7 vector between the *EcoRI* and *BamHI* sites. The recombinant plasmid was co-transformed into yeast Y1HGold with pAbAi-*GhP5CS*, pAbAi-*GhABF1*, pAbAi-*GhABF2*, pAbAi-*GhDREB2*, and pAbAi-*GhRD29*. pGADT-53 was used as a positive control, and all transformed candidates were grown on SD/-Ura/-Leu medium with 0 or 100 ng/mL of Aureobasidin A (AbA) for 3–5 days.

### 4.11. Statistical Analysis

The data were statistically analyzed using SPSS 10.0 software and plotted by GraphPad Prism 5.0. There were 3 biological replicates for each experiment, and the data were presented as means ± SD of three independent experiments. Experimental data were analyzed by Student’s *t*-test, and the bars with different letters indicate significant differences (*p* < 0.05).

## Data Availability

The data that support the findings of this study are available from the corresponding authors upon reasonable request.

## Supplementary Materials

The following supporting information can be downloaded at: https://www.mdpi.com/article/10.3390/plants13131814/s1, Table S1. WRKY genes in *G. arboreum*, *G. raimondii*, *G. hirsutum*, and *G. barbadense*. Table S2. WRKY genes containing 2 conserved motifs in *G. arboreum*, *G. raimondii*, *G. hirsutum*, and *G. barbadense*. Table S3. *cis*-acting elements in the promoter regions of WRKY genes in *G. arboreum*, *G. raimondii*, *G. hirsutum*, and *G. barbadense*. Table S4. Dispersed, segmental, tendam and proximal duplications of WRKY genes in *G. arboreum*, *G. raimondii*, *G. hirsutum*, and *G. barbadense*. Table S5. Synteny analysis of WRKY genes in *G. arboreum*, *G. raimondii*, *G. hirsutum*, and *G. barbadense*. Table. S6 The primers used in the study. Figure S1 qRT-PCR identification of *GhWRKY31* transgenic *Arabidopsis* lines. The two-week-old seedlings were used to perform the molecular identification of *GhWRKY31* transgenic *Arabidopsis* plant and lines. WT: wild type; OE1, 2, 3, 4, and 5: *GhWRKY31* transgenic *Arabidopsis* lines of T_3_ generation. The lower letter above the bar indicates the significant difference (*p* < 0.05). Figure S2 qRT-PCR identification of silencing efficiency in cotton seedings. The data represent the means ± SE from three independent experiments. Independent t-tests indicated that there was significant difference among the ‘TM1’, ‘TM1 + pYL156:00’ and ‘TM1+pYL156:*GhWRKY31*’. The lower letter above the bar indicates the significant difference (*p* < 0.05).
